# Maturity-Onset Diabetes of Young Type 5: Diabetes with Extrapancreatic Features

**DOI:** 10.1155/2021/8243471

**Published:** 2021-11-10

**Authors:** Adnan Haider, Oksana Symczyk, Ayesha Hassan, Muhammad Atif Khan, Inderpreet Madahar, Dylan Holland

**Affiliations:** ^1^600 Suncrest Towne Center, Morgantown, WV 26505, USA; ^2^Department of Endocrinology Department of Medicine, West Virginia University, Morgantown, WV, USA; ^3^Department of Internal Medicine, West Virginia University, Morgantown, WV, USA; ^4^Ohio State University, 281 W Lane Avenue, Columbus, OH 43210, USA; ^5^West Virginia School of Medicine, Morgantown, WV 26506, USA

## Abstract

**Objective:**

This case involves a new-onset diabetes patient diagnosed during pregnancy with the congenital dysplastic right kidney. *Case Report*. Clinical presentation, biochemical features, imaging in a patient with diabetes diagnosed during pregnancy, and congenital dysplastic right kidney. *Discussion*. We present a case of a 22-year-old female with the congenital dysplastic right kidney diagnosed with gestational diabetes mellitus after failing a 1-hour oral glucose tolerance test, requiring insulin during pregnancy. Because of the family history of diabetes and morphologic renal abnormalities at young ages on the maternal side of the family, our patient was evaluated for maturity-onset diabetes of adult and was found to have HNF-1*β* mutation.

**Conclusion:**

This case highlights the importance of considering the diagnosis of maturity-onset diabetes of young and particularly MODY-5 in individuals with extrapancreatic features. MODY-5 should also be considered in a patient undergoing renal transplant at young ages with a family history of morphologic renal abnormalities.

## 1. Background

MODY-5 is caused by an autosomal dominant mutation in the HNF1b gene. Our objective is to report a case of a young female with a history of the dysplastic right kidney, diagnosed with gestational diabetes. Morphologic kidney abnormalities, electrolyte abnormalities (low magnesium and high urate), strong family history of diabetes, and renal abnormalities at young ages should alert the clinician of this diagnosis.

## 2. Case Report

**Figure 1:**
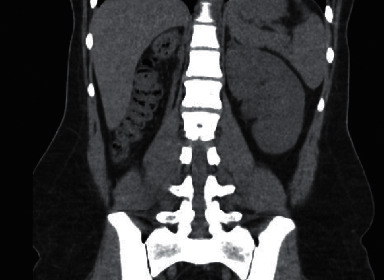
Absent right kidney with compensatory hypertrophy noted in the left kidney.

A previously healthy 22-year-old female was diagnosed with gestational diabetes after failing her 1-hour glucose tolerance test at 28 weeks gestation with a blood glucose level of 241. Elevated glucose levels were noted on random basic metabolic panels done prior to pregnancy, but the patient did not meet criteria to diagnose diabetes mellitus. Patient had no other symptoms at the time of the abnormal oral glucose tolerance test. The pertinent medical history prior to pregnancy included ultrasounds showing a small multicystic dysplastic right kidney. Patient's left kidney showed compensatory hypertrophy and a small midpole cyst (Figure 1). Kidney function is followed by nephrology and has been unremarkable. The family history is positive for polycystic kidney disease in her mother and maternal grandmother.

Due to her diagnosis of gestational diabetes, prior elevated glucose readings, and pertinent medical history, a polycystic disease panel was performed to test for mature onset diabetes of the young (MODY). This panel came back positive for a pathologic mutation to HNF-1*β* with deletion of exons 1–9 with genomic coordinates chr17 : 36047329_36105161 (GRCh37). The patient was subsequently diagnosed with MODY-5. Patient was originally managing glycemic control with diet and exercise but eventually was placed on basal insulin and titrated to meet the glycemic targets during pregnancy. Patient has required no insulin since giving birth to her baby.

**Figure 2:**
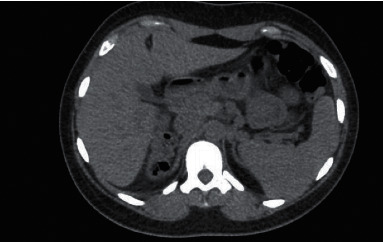
The uncinate process, head and body appear normal in size, but pancreatic tail is not visualized on noncontrast CT scan.

Patient did experience other extrapancreatic symptoms that included low magnesium (1.3 mg/dl), high uric acid (11.1 mg/dl), and pancreatic hypoplasia noted on previous CT done two years prior to her diagnosis. CT scan was done to rule out renal stones and did show pancreatic tail hypoplasia (Figure 2). Patient's most recent blood work done 6 weeks following delivery is given in Table 1. She delivered a healthy baby girl via spontaneous vaginal delivery at 39 weeks of gestation weighing 3170 g with APGAR score of 8 and 9 at 1 and 5 minutes, respectively. Her daughter has normal kidneys.

## 3. Discussion

Monogenic forms of diabetes result from a single gene defect with an autosomal dominant pattern of inheritance. A high index of suspicion based on the strong family history of diabetes at young ages in at least three generations is suggestive of this diagnosis which can affect successful patient management, ensure healthy pregnancy in female patients, and offering genetic counselling to the families.

To date, fourteen different genes have been implicated in the etiology of MODY. GCK mutation is the most common culprit mutation accounting for 30–50% of all MODY [1]. Hepatic nuclear factor 1B gene identified in patients with MODY-5 comprises less than 5% of MODY subtypes [1].

Although the exact prevalence is not known, current estimates suggest that 1–5% of all diabetes cases in the United States and other developed countries are a monogenic form of diabetes [2, 3]. Often the diagnosis is overlooked and misdiagnosed as diabetes type 1 or type 2, and therefore, the true prevalence of MODY may be higher. Given the absence of a typical phenotype and the cost of genetic analysis, it is indeed a challenge to distinguish MODY from many people with young-onset type 2 and type 1 diabetes. Patients with MODY are often misdiagnosed as type 2 diabetes, mainly if hyperglycemia was first detected in adulthood. Several reasons such as the increasing prevalence of diabetes among youths, positive family history of diabetes, lack of characteristic clinical features, and therapeutic response to insulin secretagogues add to this confusion. Similarly, if they are present in their early teens, the default diagnosis is usually type 1 diabetes, and they are put on insulin, sometimes with multiple daily injections a day. Most MODY types do not involve extrapancreatic organs; in contrast, MODY-5 frequently comprises extrapancreatic organs [4].

The first case of MODY-5 was reported in 1997 in a Japanese family [5]. The HNF-1*β* is encoded by the human transcription factor 2 gene and is expressed in a wide variety of tissues, including the kidney, pancreas, genitourinary epithelium, liver, and lung during embryogenesis [6].

HNF1B-associated MODY is a multisystem disease and includes genital tract malformation, abnormal liver function test, hypomagnesemia, and hyperuricemia associated early gout; neurological features such as autism spectrum disorders and cognitive impairment are also seen in MODY-5. Renal cysts are the most frequently detected feature of HNF-1*β*-associated kidney disease, and MODY-5 is also known as renal cysts and diabetes (RCAD) syndrome.

Patients with a strong family history of diabetes at young ages and renal morphologic abnormalities diagnosed at or soon after birth should be considered for the HNF1B screen.

Diabetes prevalence was 80% and that of renal malformations was 91% in HNF-1*β* mutation. Diabetes can present with weight loss, polyuria, and polydipsia in about half of the patients; ketoacidosis was the initial presentation in 5% of the patients. A1c profile in MODY-5 patients showed A1c ranging from <7% in one-third of the patients to >13% in another 35% of the patients at the initial diagnosis [7].

Pancreatic hypoplasia with resulting beta-cell hypoplasia results in decreased insulin secretion and release, altered glucose-sensing, and increased hepatic insulin resistance which also play a major role in the development of diabetes. Severity of pancreatic hypoplasia determines the severity of hyperglycemia and possibility of ketoacidosis at initial presentation. Imaging of the pancreas is therefore helpful in MODY-5 patients. Nongenetic factors that affect insulin sensitivity (infection, puberty, pregnancy, and rarely obesity) can trigger diabetes onset and affect the severity of hyperglycemia in MODY but do not play a significant role in the development of MODY [8].

Renal functional decline is reported; a median yearly decrease in the estimated glomerular filtration rate (eGFR) of 2.45 ml/min/1.73 m2 was observed in a study of 27 adults with an HNF-1*β* mutation and a wide variety of renal phenotypes [9]. MODY-5 patient who develops ESRD should be considered for renal transplantation; however, these patients are at increased risk of developing posttransplant diabetes mellitus [10]. Like type 1 diabetes patients requiring renal replacement therapy, HNF-1*β* patient should be considered for simultaneous pancreatic and renal transplant [10]. Functional renal anomalies notably hypomagnesemia and hyperuricemia are also seen in MODY-5 patients. HNF-1*β* transcription functions over the FXYD domain containing ion transport regulator 2 (fxyd2), a gene that encodes sodium-potassium ATPase in the distal convoluted tubule, which plays an important role in magnesium reabsorption. HNF-1*β* is also responsible for transcription of uromodulin gene which is involved in urate transport [11]. Unlike asymptomatic hypomagnesemia, elevated uric acid levels can present as symptomatic monoarticular gout. Primary hyperparathyroidism has been identified because HNF-1*β* inhibits the transcription of parathormone hormone [12].

## 4. Conclusion

MODY-5 should be considered a multisystem disorder and should be considered in the differential in young patients with renal malformations.

Another group of patients where screening for HNF-1*β* will be helpful and can change management are young patients requiring renal transplantation secondary to morphologic renal abnormalities such as renal cysts. Retrospectively screening such a population for HNF1B mutation might provide exciting information about the prevalence of this mutation.

## Figures and Tables

**Table 1:** Pertinent labs done on the patient, showing low magnesium and high uric acid levels.

Lab	Value	Normal range
Fasting glucose	113 mg/dl	<100 mg/dl
C-peptide	2.8 ng/ml	0.9–7.1 ng/ml
Magnesium	1.3 mg/dl	1.8–2.6 mg/dl
Calcium	9.9 mg/dl	8.5–10.0 mg/dl
Albumin	4.5 mg/dl	3.2–4.6 mg/dl
Uric acid	11.1 mg/dl	2.9–6.3 mg/dl
PTH	70.1 pg/ml	8.5–77 pg/ml
Creatinine	1.08 mg/dl	0.60–1.05 mg/dl
GFR	73 ml/min/BSA	<60 ml/min/BSA

## Abbreviations

MODY: Maturity onset diabetes of young
HNF-1*β*: Hepatocyte nuclear factor one beta
OGTT: Oral glucose tolerance test
GCK: Glucokinase.
